# Reducing Did Not Attends (DNAs) at a Community Mental Health Service Through the Implementation of SMS Reminders: A Closed Loop Audit

**DOI:** 10.1192/bjo.2025.10351

**Published:** 2025-06-20

**Authors:** Min San Chong, Ahmed Jawad

**Affiliations:** 1 Central and North West London NHS Foundation Trust, London, United Kingdom; 2 North London NHS Foundation Trust, London, United Kingdomm

## Abstract

**Aims:** This audit aims to ascertain the rate of Did Not Attends (DNAs) in the St John’s Wood PCN, North Westminster CMHT between 01 March 2024 to 31 May 2024 and to evaluate the rate of DNAs following the implementation of SMS reminders one working day prior to a scheduled appointment between 01 June 2024 and 31 August 2024.

**Methods:** The rate of DNAs for outpatient appointments booked between 01 March 2024 to 31 May 2024 were reviewed by scoring the number of DNAs out of the total number of appointments booked. The percentage was calculated and tabulated using Microsoft Excel for simple statistical analysis.

SMS reminders were sent one working day prior to a scheduled appointment for appointments booked from 01 June 2024 onwards. A re-audit of the rate of DNAs was conducted for the period 01 June 2024 to 31 August 2024 and the results were presented to the Multidisciplinary Team.

**Results:** The data collected reflects an overall mean improvement of 33.3% in the rate of DNAs following the implementation of SMS reminders one working day prior to a scheduled appointment. The mean percentage of DNAs decreased from 7.5 % to 5.0% following the implementation of SMS reminders one working day before a scheduled appointment.

**Conclusion:** This audit reflects an overall improvement of 33.3% in the rate of DNAs by successfully reducing the rate of DNAs from 8.3% (highest) to 4.1% (lowest) with a mean improvement of 2.5%. The intervention is deemed successful and feasible for long-term implementation within the service. We recommend a re-audit 12 months post intervention to evaluate its sustained effectiveness within the service.

